# Consumers’ Awareness, Behavior and Expectations for Food Packaging Environmental Sustainability: Influence of Socio-Demographic Characteristics

**DOI:** 10.3390/foods11162388

**Published:** 2022-08-09

**Authors:** Chiara Chirilli, Martina Molino, Luisa Torri

**Affiliations:** University of Gastronomic Sciences, Piazza Vittorio Emanuele II 9, 12042 Pollenzo, Italy

**Keywords:** consumer perception, environmentally sustainable packaging development, eco-labels, packaging information, gender effect, age effect

## Abstract

Packaging is a leading factor determining the total environmental effect of food products. This study investigated consumers’ awareness, behavior and expectations in relation to the environmental sustainability aspects of food packaging. Using an online survey, responses from 646 participants were collected. The effect of socio-demographic characteristics on all variable responses was explored by ANOVA models and *t*-tests. Participants were segmented according to a visual approach based on a principal component analysis applied on the consumers’ behavioral data. Gender, age, and education level affected consumer awareness, behavior and expectations differently. Four groups of consumers were distinguished on the grounds of their behavior in relation to food packaging: (1) More sustainable—packaging-role-oriented; (2) More sustainable—packaging minimizers; (3) Less sustainable; and (4) Medium sustainable. The most sustainable groups were mainly composed of females, while less sustainable consumers were mainly the youngest. The four groups differed in terms of expectations for sustainability-related information that can be communicated through food labels. In conclusion, this work provided new knowledge that is useful to understand the factors that influence consumer behavior and to promote the consumers’ packaging-related sustainability choices through food packaging.

## 1. Introduction

### 1.1. Environmentally Sustainability Packaging

Sustainable development, according to the European Union, is based on connecting economic, environmental and social issues to promote a growth model that can satisfy current requirements while guaranteeing and protecting the demands and needs of tomorrow [1]. Politics, academia and industry could be decisive in supporting environmental sustainability by developing circular solutions using biodegradable or bio-renewable materials to conserve resources and raw materials [2]. The concept of sustainable development can be applied to all stages of the life cycle of packaging. Packaging materials are mainly used by the food industry sector and provide safety and quality to the packaged product [3,4]. Moreover, packaging must meet communication, legal and business needs [5]. On the one hand, packaging performs a fundamental role in the protection of products, preserving the content from external effects such as air, heat and microorganisms, and protecting it from mechanical damage, representing an adequate barrier to gases and vapors, preventing food loss throughout the supply chain [6]. Packaging is a key factor in terms of sustainability, ensuring food safety by preventing the damage and perishability of goods, increasing shelf life and reducing waste, in order to protect the environment from an unnecessary increase in product and waste generation [7]. Functional and appearance characteristics, environmental impacts, costs in terms of production processes and end-of-life assessment, and effects on the local and worldwide impact are the factors that mainly condition the sustainability of a packaging material [2]. By contrast, in terms of environmental analysis, packaging has a life cycle of its own that can involve a high environmental impact and it is indeed considered a key factor in the sustainability of the food industry. Although packaging can help minimize food waste, it is responsible for an increased ecological footprint as it consumes a lot of materials, necessitates transport and involves end-of-life management [8,9].

### 1.2. Innovations for Environmentally Sustainable Food Packaging

Packaging can be called environmentally sustainable when it is possible to optimize the packaging for all phases of its life cycle; however, doing so involves compromises. An optimal solution to reduce the environmental impact could be to minimize the material used while maintaining the mechanical properties [10]. There are three factors in particular that need to be addressed in obtaining environmentally sustainable packaging [11]: (i) raw materials, preferring recycled and renewable resources in order to decrease CO_2_ emissions and the consumption of fossil energy resources; (ii) making processes more energy efficient; and (iii) focusing on reuse, recycling and biodegradation for improved waste handling. Azzi et al. [12] discovered that, on average, 9% of the final product value is related to packaging costs, while 60% of the total cost of producing packaging is spent on the use and disposal of the material. Furthermore, concerning grocery products, 65% of the worldwide domestic waste generated is packaging materials. For this reason, there are many studies promoting the development of environmentally sustainable packaging solutions. On the one hand, one of the main goals is to decrease the impact of packaging by resorting to solutions that prefer bio-based materials, promoting the efficiency of packaging use and improving the performance of components and their subsequent recycling by reusing a post-consumption material or reusable packaging [2,13]. Conversely, when developing alternative packaging, the attention on increasing the quality profile of the packaged goods and reducing the possibility of food becoming waste is not neglected by devising solutions that could extend the durability of packaged product [13]. Therefore, research is moving towards new preservation techniques, such as active and bioactive packaging and smart packaging, which can intentionally interact with food and the surrounding environment providing consumer health benefits [14,15].

Consumer preferences for food products that are both shelf-stable and convenient, modern trends in retail practices and changing lifestyles are the issues that have led and driven us toward new and advanced packaging technologies that do not compromise consumer health and food security [4].

### 1.3. The Role of Consumers in Promoting Environmentally Sustainable Packaging

Packaging properties alone are not enough to provide an environmental benefit to the sustainable packaging itself but must be assisted by consumers’ desire to purchase sustainable products. Environmental issues related to packaging are now in the public domain, affecting the entire population as well as institutions and industries, thus fostering initiatives to devise sustainable packaging designs [16]. Therefore, consumers can play a key role in promoting the demand for an increased use of bio-based materials as a suitable alternative to materials produced from non-renewable resources. In addition, it is consumer demands that drive companies to innovate in packaging so that they can pander to and predict new global trends such as increased life expectancy or diversified food distribution [17].

There are multiple factors that drive purchasing choices and the consequent consumption of products, starting from the individual and demographic characteristics of consumers, marketing strategies and price, to environmental consciousness, but not underestimating the attributes of packaging, meaning, design, aesthetics, functionality and the amount of product it can contain [18,19]. Therefore, to meet the multiple demands of consumers, packaging must be constantly innovated and improved. Since environmental knowledge is also a factor that can currently drive consumers to increase their purchase of green products, it is inferred that environmental knowledge may have a significant and positive influence on consumers’ ecological behavior. [20]. However, such knowledge could make consumers aware of the shortcomings that the market offers, limiting purchases [7].

The challenge, in order to achieve real environmental improvements, is to convince consumers to move away from conventionally packaged products and instead to prefer sustainably packaged products. Much research confirms that consumers are moving in this direction [7,21] arguing that environmentally sustainable packaging is now a factor influencing buyers’ consumption preferences and readiness to pay [16]. However, the concept of environmental sustainability is not always connected to positive connotations, negatively influencing consumer satisfaction and perception. For instance, the idea of environmental sustainability may be related to the idea of a lack of product functions and users are not willing to compromise practical efficiency for ethical values such as environmental sustainability [22]. Therefore, the idea that redesigning packaging to increase environmental sustainability is synonymous with less functionality may negatively influence the likelihood of purchasing. In addition, the concept of environmental sustainability is also often linked to the idea of higher production costs, so the ability to pay more could preclude people from making ecological choices.

Perceived environmental sustainability is most often related to sensed genuineness and ethical gratification, which are factors that can contribute to consumers’ likelihood to purchase [16]. However, although aspects such as “healthiness” and “environmental friendliness” are attributes that are considered by consumers at the time of purchase, they may not automatically be reflected into their concrete action [23]. Indeed, understanding consumers’ behavior is a necessary prerequisite for effective marketing [24]. Therefore, companies could help people make healthy and environmentally friendly choices by communicating these issues more effectively and truthfully through packaging design, packaging images and labels. Better communication by companies willing to take on their social responsibility could encourage consumers to make greener and more environmentally sustainable behaviors and choices. Food labels are an important information vehicle for consumers by providing both details about ingredients and allergens, and health or environmental effects, and food label designers play a key role in how this voluntary information is communicated [23].

### 1.4. The Influence of Socio-Demographic Characteristics on Consumers’ Environmental Concern and Sustainable Packaging Perception

While consumer attitudes toward packaging design is a topic extensively covered in the literature, there are few studies that consider the effect of sustainable packaging on consumer choices [25]. However, several studies examined socio-demographic characteristics, such as gender, age and education level, to analyze environmentally sensitive consumers, yielding different results. The literature review by Ketelsen et al. [26], for example, showed the influence of demographic characteristics (sex, age and education level) of consumers on their response to green packaging, showing that women were much more interested in the decomposition time of packaging and minimizing packaging waste; however, men were more sensitive to the environmental aspects of packaging than women. In addition, older participants were more interested in packaging materials and their environmental impact and were more likely to pay more for environmentally friendly packaging. Finally, people with a higher level of education paid more attention to the type of packaging material.

In addition, regarding gender, many studies have shown that females are much more environmentally conscious than males and are concerned about the environmental impact that their actions and consumption may have [27,28,29]. The purchasing decisions of females are much more driven and determined by environmental considerations than those of males [30] and are positively influenced by eco-friendly labeled products [31]. In addition, Martinho et al. [7] reported that females preferred eco-friendly packaging more than males. 

Age is another demographic variable that has been much examined in past studies, although the results are somewhat inconsistent. Indeed, some studies [27,32,33] have revealed a positive relationship between age and environmental concern and recycling behavior, while other studies have shown that younger consumers have a strong awareness of the need for environmental preservation and are more involved in sustainable issues [34].

In previous works, consumers’ level of education is regarded as a socio-demographic factor that can influence their environmental choices. Indeed, several studies have shown that more educated people are more aware of environmental issues and sensitive to the quality of the environment and, as a result, have a greater propensity to engage in green consumption behaviors [28,33]. People with a high level of education tend to purchase sustainable products more often [34] and individuals with a high level of education are more likely to purchase green products in the presence of eco-labels [31].

In this scenario, it has been hypothesized that: H1, Socio-demographic characteristics (gender, age, and educational level) influence consumers’ environmental awareness, behavior and expectations related to food packaging; H2, Segments of participants with more or less sustainable behavior related to food packaging can be identified and discriminated in terms of characteristics and attitudes. Therefore, the goals of the present research were to investigate consumers’ awareness, behavior and expectations in relation to the environmental sustainability aspects of food packaging and understand how companies could better convey concepts related to environmental sustainability through food packaging, bringing consumers closer to these issues and contributing to an improvement in environmentally sustainable development. 

## 2. Methods

### 2.1. Online Survey

A self-reported online survey consisting of 20 questions was developed employing Qualtrics^®^ software (Provo, UT) to explore consumers’ perception of environmentally sustainable issues related to food packaging. The survey was designed in Italian and subjects were recruited through a convenience sampling technique, disseminating the survey via links and a QR code via email and social media (e.g., Facebook, LinkedIn and WhatsApp). The use of social media as a method of recruiting participants has proven to be effective, as has already been shown in other studies for Facebook [35,36], as it enables large-scale data collection in a short period of time and without cost. It also facilitated heterogeneity in the data collected, with participants varying in gender, age, education level and geographic location. Data were collected anonymously over two weeks. The online study was organized in sections (see Supplementary Material). The first one consisted of questions on socio-demographic characteristics (gender, age, nationality and educational level). The other three parts, described below, included both questions deduced from the literature and questions developed specifically for this survey. The statements of the last three parts of the survey were randomized across subjects and scored on a 7-point Likert scale (1 = extremely disagree; 7 = extremely agree). The survey required approximately 10–20 min to be completed.

#### 2.1.1. Consumers’ Awareness

The second part of the questionnaire was aimed at investigating how much consumers were aware about several environmental-sustainability-related concepts, such as a circular economy, food waste and characteristics that distinguish environmentally sustainable packaging. Firstly, to assess the extent to which consumers are aware of the meaning of eco-labels on packaging, participants were asked to observe seven symbols frequently reported on food packaging labels (green Dot, universal recycling symbol, resin identification codes, Seedling^®^ compostable label, Forest Stewardship Council (FSC) label, Cradle to cradle^®^ certification label, pitch-in symbol/do not litter) and to identify them selecting the appropriate definition among six options. The symbols presented to the participants were selected based on previous research on the environmental sustainability of liquid food packaging [37]. Secondly, as was previously carried out by Lindh et al. [38], participants were requested to express their agreement with the following four statements: (1) “I am aware of the concept of circular economy”; (2) “I am aware of the concept of food waste”; (3) “I think that it is important to consider packaging material when purchasing”; (4) “I think that the food product packaging and food waste are related”. Similarly, respondents were asked to rate their level of agreement with five statements related to the characteristics that make packaging environmentally sustainable, accordingly to Lindh et al. and Korhonen et al. [38,39]: (1) “Use of nanotechnologies”; (2) “Made by regenerated materials”; (3) “Smart/active function”; (4) “Packaging reduction”; (5) “Produces no waste and is 100% reusable”.

#### 2.1.2. Consumers’ Behavior

The third part was developed to understand consumers’ behavior in terms of daily life choices related to food packaging and environmental sustainability. Participants were required to state their level of agreement with five statements that describe their behavior, as reported by Korhonen et al. [39]: “I buy products in bulk”; “I try to buy products that have less packaging”; “I reuse the packaging of the products I buy”; “I prefer to buy products whose packaging allows a longer shelf life”; “I read the description of the packaging”. Similarly, subjects were asked to score the statements specifically developed for this survey “I reduce the purchase of food in plastic packaging”, “I pay attention to separate waste collection”; “I usually buy products from companies whose environmental sustainability values I know” and “I reduce food waste”.

#### 2.1.3. Consumers’ Expectations

The fourth part of the questionnaire was developed to investigate how important consumer evaluation is of the environmental-sustainability-related information that can be reported on a food packaging and what they expect from food companies to improve communication regarding the environmental sustainability characteristics of the packaging. Respondents were asked to state their level of agreement with a range of eight statements, in agreement with Martinho et al. [7], regarding what information they thought was important to find on food packaging: “Indication of packaging material”; “Indication of the type of collection”; “Symbols relating to the environmental sustainability of the packaging”; “Narrative elements that tell the type of the packaging”; “Packaging ecological footprint”; “Country of origin of the food product”; “Nutritional values of the food product”; and “Expiration date of the food product”. Similarly, participants were required to rate their level of agreement with a range of six statements specifically developed for this survey (“Clearer and larger symbols”; “Description of symbols”; “Environmental impact phrases”; “More details about the materials that make up the packaging”; “More details on how to recycle”; and “QR codes or digital tools”) completing the following sentence: “I think that companies could increase the communication of the food packaging sustainability, improving the label by means of …”.

### 2.2. Participants

A total of 1018 subjects had access to the survey, but 372 did not complete it entirely. Out of the 646 participants who completed the responses, 96% declared an Italian nationality, 63.5% were females (*n* = 410), 35.1% (*n* = 227) were males, 0.2% (*n* = 1) listed “other gender”, and 1.2% (*n* = 8) preferred not to report. Given that subjects declaring “other gender” and preferring not to report were insufficient to allow a reliable comparison among gender groups, they were not included in the analysis conducted to evaluate the gender effect. Thus, further statistical analysis on gender effect was conducted on a sample of 637 subjects (64.4% females; 35.6% males). The age of participants ranged from 18 to 80 years, with a mean age of 38.5 years and a standard deviation of 15.0 years. To investigate the age effect on responses, participants were classified into four age groups: 18–30 years (*n* = 272, 42.1%), 31–45 years (*n* = 128, 19.8%), 46–60 years (*n* = 191, 29.6%), and 61–80 years (*n* = 55, 8.5%). Participants were also grouped into four categories based on their level of education: the first included participants who had only a primary or lower secondary school license (*n* = 32.5%); the second included those who had an upper secondary school diploma (*n* = 227, 35.1%); those who had a bachelor’s degree were part of the third category (*n* = 153, 23.7%); and the fourth included those who had a master’s degree, a post-degree or a doctoral degree (*n* = 234, 36.2%).

All subjects provided informed consent. The study was approved by the Ethics Committee of UNISG (Ethics Committee Proceeding n. 2022.01). The research was conducted in accordance with the Declaration of Helsinki.

### 2.3. Data Analysis

Three two-way ANOVA mixed models (fixed factor: variable item; random factor: subject) were independently applied to the three datasets obtained from all 646 participants regarding consumers’ responses in terms of awareness, behavior and expectations to evaluate the effect of the variable items. 

The *t*-tests were conducted to assess the gender effect (females vs. males) on all items in the three datasets related to consumer awareness, behavior, and environmental expectations. One-way ANOVA models were independently applied to determine the main effects of age (four levels: 18–30, 31–45, 46–60, and 61–80) and educational level (four levels: primary/lower secondary school, upper secondary school, bachelor’s degree, and master’s/post/doctoral degree) on consumers’ awareness, behavior and expectations.

Consumers’ behavior in relation to food packaging data (variables: seven items) expressed by all 646 participants (observations) were analyzed by means of a principal component analysis (PCA). PCA was chosen to obtain a biplot representing a map able to show the distribution of the subjects based on their sustainable behavior related to food packaging. According to a visually oriented approach, the biplot obtained from the PCA allowed the segmentation of the participants into four groups based on their distribution in the four quadrants. The four groups identified by the biplot represent four different styles of behavior toward food packaging. One-way ANOVA models were applied to estimate the effect of the four identified groups on all investigated variables related to awareness, behavior and expectations. Two-way ANOVA mixed models (fixed factor: variable item; random factor: subject) were independently applied to the data of each group of participants to investigate the effect of the items of all three types of variables (awareness, behavior and expectations) within the groups. The distribution of participants in the four groups according to gender, age and education level was assessed by chi-squared tests to understand if the four groups with different behavior were significantly differently composed in terms of socio-demographic characteristics.

All ANOVA models were followed by a Tukey’s HSD test (alpha = 0.05). All analyses were performed using the XLSTAT statistical software package version 2022.1.2 (Addinsoft, New York, NY, USA).

## 3. Results

### 3.1. Consumers’ Awareness of Environmental Packaging Sustainability-Related Concepts

Considering the responses obtained from the totality of the participants, the two-way ANOVA results showed significant differences (F = 1295.80, *p* < 0.0001) (Table 1) among the items related to what was known by consumers about the packaging sustainability-related concepts. In particular, the highest mean values were observed for the importance to consider the packaging material when purchasing and for the awareness of the concept of food waste, while a significantly lower mean awareness was found for the concept of a circular economy. Even lower was the mean value observed for the relationship between packaging and food waste. The lowest mean value was noticed for the knowledge of the eco-symbols used for packaging. A significant clear discrimination (*p* < 0.0001) among the items regarding what participants think makes packaging sustainable was observed with mean values of “Produces no waste and is 100% reusable” > “Packaging reduction” > “Regenerated materials” > “Smart/active function” > “Nanotechnologies”.

Consumers’ awareness of environmental-sustainability-related concepts was influenced by the investigated socio-demographic characteristics. The *t*-test carried out to examine the gender effect on what the participants know about sustainability-related concepts revealed a significant difference for two items. In particular, females declared a significantly greater awareness of the concept of “food waste” than males (*p* = 0.012) and they believed it is relevant to take into account the material of the packaging when purchasing more than males (*p* = 0.002). Regarding what participants think makes packaging sustainable, there were two significant differences between the two groups, with females believing more than males that the characteristics that can make packaging sustainable are the active/intelligent function (*p* = 0.033) and that it is made by reducing the packaging material used (*p* = 0.047). The ANOVA results showed a limited effect of the age on consumer awareness for environmental-sustainability-related concepts. The only significant difference due to age class was found for the knowledge of the meanings of symbols that can be found on the label of food packaging (F = 3.16, *p* = 0.024), with the oldest participants (61–80 years) turning out to be less informed than participants aged 18–45 years old. A significant effect of the educational level on consumers’ awareness was found from the ANOVA results for several variables. For participants with the lowest level of education (primary school/lower secondary school), the significant lowest mean values were observed for all items investigating what participants knew about environmental sustainability (*p* < 0.05), except for the item relating to packaging and the food waste. Moreover, participants with the highest educational level (master’s degree/post-degree/doctoral degree) provided a significantly higher mean value than those with an upper secondary school level of education for the knowledge of the symbols and of the concept of food waste. 

### 3.2. Consumers’ Behavior in Relation to Food Packaging

The mean values of the scores provided by all participants for the items regarding the consumers’ behavior in relation to food packaging are reported in Table 2. The results of the two-way ANOVA followed by the Tukey’s test showed a significant effect of the item on consumers’ responses (F = 187.35, *p* < 0.0001). The highest-scored environmentally sustainable behaviors were those regarding waste (“I pay attention to separate waste collection” and “I reduce food waste”), followed by the items related to a reduction in the packaging material (“I try to buy products that have less packaging” and “I reduce the purchase of food in plastic packaging”) and by the items implying a reuse or avoidance of the packaging (“I reuse the packaging of the products I buy” and “I reuse the packaging of the products I buy”). The least adopted behaviors were the habits to buy products whose packaging allows a longer shelf life and products from companies whose environmental sustainability values are known.

Consumers’ behavior in relation to food packaging was also influenced by the investigated socio-demographic characteristics. Gender clearly discriminated the consumers’ behavior in relation to food packaging in several terms: compared to males, females were significantly (*p* < 0.05) more inclined to buy products in bulk or with less packaging, to reduce the purchase of plastic, to reuse packaging and to pay more attention to separate waste collection. On the contrary, no evident effect of age on behavior was found. The only significant differences were found for two items: the 31–45 age group reduced the purchase of food in plastic packages less than the 61–80 age group (F = 4.01, *p* = 0.008), while the 46–60 age group paid more attention to separate waste collection than the youngest group (18–30 years old) (F = 3.66, *p* = 0.012). Educational qualification did not seem to have a relevant effect on subjects’ behavioral choices. Indeed, there was only one significant difference (F = 3.73, *p* = 0.011) indicating that participants with the highest educational level were more careful about separating waste collection than those with the lowest level of education.

### 3.3. Importance of and Expectations for Environmental-Sustainability-Related Information on Food Packaging

The mean values associated with the types of information considered important to be on the labels (Table 3) revealed that consumers paid significantly (F = 189.75, *p* < 0.0001) more attention to the characteristics of the product, such as the expiration date, the nutritional values and the country of origin, than the information related to the packaging. Among the latter, the most important information was the indication of the type of collection, followed by the indication of the packaging materials and symbols related to the environmental sustainability of the packaging. Less important were the packaging ecological footprint and the narrative elements that indicate the type of packaging. Regarding the expectations for eco-labels, the consumers would like to find more details on how to recycle the packaging and about the materials that made the packaging. Moreover, they would like better described, clearer and larger symbols. A significantly lower interest was observed towards QR codes or digital tools and environmental impact phrases.

The results regarding consumer evaluation of the importance of and expectations for environmental-sustainability-related information on food packaging were affected by the socio-demographic characteristics. A significant gender effect was found to affect what consumers think is important to be reported on the labels. In particular, females believed that it was more important than males to find narrative elements that indicate the type of packaging (*p* = 0.042) and information on how to separate packaging waste for collection (*p* = 0.019), information on the country of origin of the food product (*p* = 0.026) and information regarding the packaging ecological footprint (*p* = 0.044). Similarly, females were more of the opinion than males that eco-labels could be improved through a better description of the eco-symbols reported on packaging (*p* = 0.024) and with more details on how to recycle the packaging (0.004). Age did not have a strong influence on what is important to find on labels. Even if a trend in the responses as a function of the age class was not evident, a significant difference was observed for two items. Firstly, the youngest group (18–30 years old) considered it less important than the 46–60 year age group to read narrative elements on the labels that indicate the type of packaging (F = 3.76, *p* = 0.011). Secondly, this last group considered it more important than the 31–45 year age group to find the expiration date of the food product on the packaging (F = 3.70, *p* = 0.012). Conversely, a clear effect of age was observed on how the eco-labels can be implemented by companies to improve communication with consumers. In fact, the 46–60 and 61–80 year age groups would like the symbols related to the sustainability of the packaging to be clearer and bigger, and to find more phrases about the environmental impact on labels, compared to the other two age groups (18–30 years; 31–45 years) (F = 11.94, 16.39, *p* < 0.0001). Considering the educational level, for participants who attended primary school or lower secondary school, it was less important to have information on collection methods on the packaging label, compared to all of the other participants (F = 6.75, *p* = 0.0002). Moreover, for them it was less important to find the indication of the packaging materials (F = 3.65, *p* = 0.012) and nutritional values (F = 4.02, *p* = 0.007), compared to the most educated subjects (having at least a master’s degree). In contrast, among the educational level groups, there were no significant differences on how they would like companies to increase communication of the concept of environmentally sustainable packaging through labelling.

### 3.4. Participants’ Segmentation

**Figure 1:**
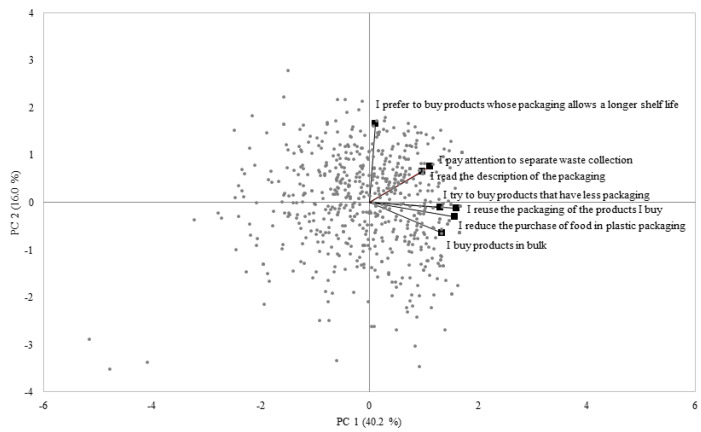
Biplot from the principal component analysis applied to the consumers’ behavior in relation to food packaging data (*n* = 646).

The biplot obtained from the PCA applied to consumer behavior in relation to food packaging data (seven items) is shown in Figure 1. The total explained variance accounted for 56.2%, with the first (PC1) and second (PC2) principal components explaining 40.2% and 16.0%, respectively. Those participants positively correlated with PC1 declared, in general, a more environmentally sustainable behavior in relation to food packaging; conversely, those who were negatively correlated provided lower scores for the items used to investigate the environmentally sustainable-related behavior of the packaging. On the other hand, the position of the participants along the positive values of PC2 was mainly influenced by the three variables: “I prefer to buy products whose packaging allows a longer shelf life”, “I pay attention to separate waste collection” and “I read the description of the packaging”. According to the visually oriented approach used for segmenting participants, four groups were identified based on their position in the four quadrants. Participants who behave more sustainably in relation to packaging are positioned in the first two quadrants. In particular, the first quadrant contains participants who are most attentive to the role and use of the packaging (Group 1; *n* = 182, 28.2%), being more prone to preferring to buy products whose packaging allows a longer shelf life, paying attention to waste separation, and reading the packaging description. In the second quadrant participants who tend to reduce packaging are found (Group 2; *n* = 169, 26.2%). In fact, they tend to reuse the packaging of the products they buy, they buy products that have less packaging, they reduce the purchase of food contained in plastic packaging and they buy products in bulk. On the contrary, in the opposite quadrants the participants who behave less sustainably in relation to packaging are positioned. In particular, the third quadrant (Group 3; *n* = 137, 21.2%) displays participants who pay the least attention to the role and use of the packaging, while the fourth quadrant (Group 4; *n* = 158, 24.5%) shows the participants who are less interested in minimizing their use of packaging.

Overall, groups 1, 2, 3 and 4 could represent four distinct segments of subjects with different environmentally sustainable packaging-related behavior, namely “More sustainable—packaging role oriented”, “More sustainable—packaging minimizers”, “Less sustainable”, and “Medium sustainable”, respectively.

### 3.5. Characterization of the Consumer Groups

The four groups of participants identified based on their packaging-related behavior were characterized as a function of all analyzed variables. Table 4 reports the results of the ANOVA performed to evaluate the effect of the group on the awareness, behavior and expectation items. Except for the awareness of the food waste concept (*p* = 0.247), all items were significantly discriminated among the four groups (*p* = 0.0002 for the symbol index, *p* < 0.0001 for all other items).

Group 1 and 2 significantly differed for five items regarding the environmentally sustainable behavior out of the seven used to segment the participants. However, Groups 1 and 2 did not differ for the two behavior items unrelated to packaging that were not used for participants’ segmentation (“I usually buy products form companies who environmental sustainability values I know” and “I reduce food waste”), nor for items concerning awareness (what they know and what they think makes packaging sustainable) and those regarding what is important to be on labels or how the eco-label could be improved. Groups 3 and 4 showed many significant differences compared to Groups 1 and 2, not only in terms of behavior related to packaging, but also for the awareness and expectation items.

The third group provided the lowest mean values for all significant attributes discriminating between Groups 3 and 4, suggesting the lowest attitude to make choices and actions aimed at environmental sustainability. In particular, in terms of awareness, compared with the others, Group 3 considered the type of packaging material at the time of purchase to be less important, and less agreed that the use of regenerated materials or environmentally friendly packaging makes packaging environmentally sustainable. Regarding the packaging-related behavior, Group 3 paid the least attention to separating waste and reducing food waste. Moreover, this group considered it less important to find indications regarding the packaging material and the type of collection, narrative elements that indicate the type of the packaging and information on the packaging ecological footprint on the labels. Coherently, Group 3 agreed the least that companies could increase the communication of the food packaging environmental sustainability improving the label by means of clearer and larger symbols, the description of symbols and the environmental impact phrases, and including more details about the materials that make up the packaging. Generally, Group 4 had intermediate mean values between the first two segments and the third segment for most of the evaluated items. Moreover, this segment had a higher awareness, higher environmentally sustainable behavior and more expectations than Group 3, except for the item “I buy products in bulk”, for which Group 3 scored higher.

The results of the two-way ANOVA models independently applied to the data of each group of participants showed a significant effect of the items (*p* < 0.0001) for all four groups and all three types of variables (awareness, behavior and expectations). However, the observed significant differences (Table 4; see the capital letters associated with the mean values within columns) among items in each group reveal that the trend of the mean values is similar to the general trend found for the totality of the participants (Table 1, Table 2 and Table 3). The only noteworthy difference was that, compared to the other segments of participants, Group 3 considered QR codes or digital tools as more suitable tools available for food companies to improve the eco-labels than the use of phrases regarding the environmental impact of the packaging. 

The results of the chi-squared tests and Fisher’s exact probability tests allowed identification of the significant differences among the four groups according to their socio-demographic characteristics (Table 5). The two groups of participants acting more environmentally sustainably towards food packaging (groups 1 and 2) were composed of a significantly higher proportion of females than males, compared to the two groups of participants declaring a lower environmentally sustainable behavior (groups 3 and 4). Moreover, Group 2 included a significantly lower proportion of young participants (18–30 years old), whereas Group 3 was made up of a higher proportion of the youngest age group. No significant differences were observed in terms of distribution of the participants among the four groups as a function of their educational level.

## 4. Discussion

### 4.1. Socio-Demographic Effects on Consumers’ Awareness, Behavior and Expectations Related to Food Packaging

Socio-demographic characteristics significantly influenced environmental sustainability awareness and behavior, and shaped consumers’ expectations.

Gender had a strong effect on interest in the environment and sustainability, and on consumer behavior, showing that females were more aware of environmental sustainability issues and more careful about making environmentally sustainable choices. This is in accordance with some earlier research showing that females showed more concern for the environment than males [28,29,40]. In particular, females were more interested in the composition of the product at the time of buying, according to Lindh, Olsson, et al., [38], and were very attentive to the use of packaging and separate collection, compared to males. This result confirmed a previous study showing that females with an average age of 50 years preferred eco-friendly packaging more than males [7].

Moreover, females paid more attention to separate waste collection. This result is partially in line with the study by Oztekin et al. [41], which showed a greater innate propensity of women to recycle, even if this does not directly translate into actual intentions. Several studies [42,43] confirm this propensity, believing that this is perhaps because women are more environmentally conscious and enact more pro-environmental behaviors involving the management of daily household activities, a greater sense of responsibility to care for others by taking an altruistic and cooperative role, as well as responding more strongly to food security events [44]. Indeed, females wanted more information on labels regarding the separate collection and recycling methods. Therefore, knowing the desires of females with regard to what to find on labels could be a very important factor for companies to consider, and thus, it is critical to recognize that female gender may be responsible for increasing the purchase of ecologically labeled eco-friendly products [31]. Overall, our findings disagree with the study of Peters-Texeira and Badrie [45] who stated that gender has no influence on food packaging reactions and food preference.

Age was also a determining factor that influenced consumers on certain aspects related to environmental sustainability. Older people were less informed about the meaning of eco-symbols found on labels than younger consumers, but they were also the ones who would like more information on labels regarding the environmental impact and packaging materials. It could be hypothesized that older consumers want more information from labels than younger consumers do because it may be the most common and convenient source of information for them, while younger consumers would probably more easily access other sources of information, such as websites and technology platforms. Moreover, older consumers were also more attentive to separate waste collection than younger consumers were. This finding may partially support previous research reporting that aging consumers believed that the ability to recycle is one of the most important characteristics of packaging [32] and that aging consumers tend to be more involved in environmentally friendly and recycling behaviors. It was hypothesized that this positive age–green consumer behavior bond is linked to the “Depression-era” conservation ethic of the past generations [33]. However, the finding that younger people were less likely to recycle is partially at odds with [21], in that young Indian consumers have a strong ambient awareness and concern for protecting the environment, with Jadernà and Volfovà [34] showing that young people are more interested in sustainable aspects than older generations.

Predictably, education level influenced consumers’ awareness of environmental sustainability concepts highlighting that better grades of education bring better information and awareness, according to Susanty et al. [28], and a higher preferences for environmental protection. Furthermore, it was reported that more educated individuals would generally be more likely to perform highly in all aspects of the green consumption field (knowledge and attitudes), perhaps because complex environmental issues may be better understood by more educated people. This leads to a greater concern for environmental quality and therefore a greater propensity to engage in green consumption behaviors [33]. In fact, as shown by Jadernà and Volfovà [34], people with a high level of education demonstrate a more favorable attitude toward sustainability in retail. They identify more features of sustainability and buy sustainable products more often. For people with a high level of education, sustainable products are related to eco-friendliness and they recognize sustainable products mainly through certificates. People who are better aware are those who are conscious of environmental issues and aware of the possible repercussions of uncaring behavior, and they may have stronger environmental attitudes and seek to change their behavior for the benefit of future generations. Knowledge and awareness of environmental protection have become an important influence on consumer attitudes toward environmentally friendly products. [46,47]. Education level slightly influenced what consumers’ think is important to be on labels; this is in disagreement with Zeynalova and Namazova, who instead states that a higher level of education leads to greater interest in the information on the label [48]. Only two significant differences were observed in the indication of packaging material and type of recycling collection, whereby the least educated people were found to be the least interested. This lack of interest is consistent with them also being the people who pay the least attention to recycling collection. No significant differences were observed due to the educational level regarding the importance of the symbols relating to the sustainability of the packaging and how the eco-labels could be improved. This result is in disagreement with the previous research reporting that highly educated subjects tend to report a greater correlation between the eco-label and their willingness to buy green products, compared to less educated consumers, and suggesting the possible impact of knowledge on eco-label acceptance and confidence, which could enhance and improve their intent to purchase eco-friendly products [31].

### 4.2. Relationship among Awareness, Behavior and Expectations

Some previous researches [25,26,49,50] have considered that there is a gap between attitude and behavior: a favorable consumer attitude does not necessarily translate into effective green behavior. Therefore, it is not an assumed assumption that positive consumer attitudes regarding green products result in effective action. Accordingly, Rokka and Uusitalo [18] stated that even consumers who are found to be very knowledgeable about environmental issues are not always inclined to buy eco-friendly products. Additionally, despite consumers’ good intentions, ethical concerns are not sufficient to influence actual purchasing behavior, or, even if consumers are conscious of ethics, their environmental concerns or attitudinal factors are unable to definitively delineate ecological buying behavior [51]. In order to purchase sustainable and environmentally friendly products, it is important that consumers have the proper knowledge or are willing to inquire about it. Therefore, in order to delineate the profile of consumers, and to understand how to predict their actions and direct them towards more environmentally sustainable choices to narrow the “attitude–behavior gap”, it is necessary to investigate and examine their behaviors to determine the various reasons behind their inconsistent behavior [26].

The results of the present study outlined four consumer behavior profiles. Two of these describe consumers with environmentally sustainable behavior, demonstrating this in their daily actions. Consumers who behave environmentally sustainably are the most aware about environmental sustainability, indicating that there is a strong connection between knowledge and behavior. This is in accordance with Orzan et al. [49], who tried to explain the impact of ambient consciousness on consumers’ green behavior by suggesting that environmental awareness mitigated the association between green attitude and green behavior. Moreover, our results highlighted the importance for environmentally sustainable consumers to find information about the packaging and its environmental sustainability on the label. They have shown a lot of interest in environmentally sustainable labels, so the packaging can be an important vehicle of information and knowledge for consumers. This agrees with a previous study [49] regarding the information that should be shared to help change consumer attitudes and behavior related to environmental sustainability. That research reported that the respondents thought it was relevant to present the benefits of environmentally friendly packaging, ways to recycle different materials, the timing of decomposition and the environmental burden of packaging, and the long-term impact that non-environmental packaging has on the environment and human health. Thus, it seems crucial for companies to implement and improve information regarding packaging on food labels in order to approach and retain consumers who prefer to buy products from companies whose environmental sustainability values they know and share; this could enhance the perception of the quality of green products and increase green confidence levels between consumers [52].

The other two identified groups of consumers included people who are less inclined to make environmentally sustainable packaging-related choices and who are less attentive to the use of packaging. In this case, the less environmentally sustainable behavior is matched by a lack of awareness, knowledge and interest in environmental sustainability issues. Increased awareness of the effects of irresponsible consumption could convince consumers to implement green and more environmentally sustainable consumption. Current methods of information dissemination may be ineffective in convincing consumers of the advantages (individual and environmental) of consuming green products [25]. Labels could be a suitable tool to communicate this information by emphasizing the factors that make the product environmentally sustainable. However, the effectiveness of labels cannot be taken for granted; in fact, although consumers have paid increased attention to environmental sustainability concerns, they have limited knowledge of sustainability labels. The logos and statements are not always clearly understood and their credibility is not always clear or there are uncertainties about which authority is responsible for certification. Such misunderstanding can lead to difficulties in distinguishing them, generating confusion, reticence and disbelief on the part of consumers [53,54]. Indeed, the findings of this research reveal that consumers who behave in a less environmentally sustainable way do not pay much attention to the information on the labels; in particular, for the “Less sustainable” group, a lower interest in the information on the labels and on how to improve it was observed. The information that seems to be relatively more interesting for this category of people concerns the presence of environmental sustainability-related symbols on the packaging and the description of the symbols. This low interest for information on labels could be partially explained by the fact that the environmental sustainability characteristics of packaging are not always communicated in a suitable way to the consumers. The results of the literature review by Ketelsen et al. [26] showed that, although many people reported that they knew how to recognize environmentally friendly packaging and relied on the claims and symbols to identify it, at the same time, consumers reported that their perception of the environmentally friendly nature of packaging was shaped by the packaging design elements such as the color and images of nature This is quite alarming because it implies that consumers can be easily misled by packaging design. Indeed, previous surveys revealed that consumers were unable to identify sustainable packaging or were not sufficiently aware of what they entailed [38,55]. Therefore, the environmental sustainability properties of food packaging should be communicated clearly and meaningfully to consumers in order to be understood and considered to change behavior. The nexus between environmental sustainability information and consumer behavior is an important way to bring consumers closer to environmental sustainability, to help them make environmentally sustainable choices, and this needs to be consolidated. Despite some differences, both groups of consumers seem to appreciate the presence of symbols relating to the environmental sustainability of packaging and their description, according to previous studies revealing that consumers usually search for information that is simple and easy to use when buying green products [56]. Therefore, the presence on the packaging of simple and impactful phrases on green consumption could be a solution to inform consumers and bring them closer to environmentally sustainable choices. Consequently, the combination of appropriate labels, increased visibility and availability, and increased media could provide a means of disseminating information related to environmental questions, and might support consumers in acknowledging eco-labels and properly interpretating them [53,57]. Overall, companies should take into account the differences among consumers’ segments and their needs and expectations so that they can retain even the least interested and most skeptical consumers.

### 4.3. Limitations

The fact that this study was carried out with participants recruited through a convenience sampling technique emerged as a major limitation. The survey was available online so anyone could participate, which did not allow control of the socio-demographic characteristics of respondents. In fact, there is a disparity among age groups, since young people (18–30 years) were found to be the predominant participants (42.1%). Moreover, this percentage is not representative of the Italian population, which according to ISTAT [58] data consists of 13.2% percent of young people. This higher participation may be due to the fact that social media was used as a means of recruitment, which is more usable by young people than by the elderly. Therefore, the obtained results may not be fully transferable to the population living in Italy. It is likely that, with a more controlled recruitment method, different results could have been obtained. In addition, by recruiting respondents with a convenience sampling technique, subjects who are more attentive to the environmental sustainability aspects of packaging, even if only out of curiosity or interest, were more likely to have participated than those who are not interested in these issues. This situation may have resulted in an overestimation of positive responses in terms of awareness, behavior and expectations.

## 5. Conclusions

This study explored which factors most influence consumers in their behavioral choices and how packaging, through labelling, can communicate environmental-sustainability-related information able to drive consumers towards more environmentally sustainable choices. The results showed that gender, age and education level were significant in shaping consumers’ consideration of and expectations for environmentally sustainable packaging. The identification of four consumer profiles based on their environmentally sustainable packaging-related behaviors revealed the existence of an interrelation between the consumer behavior and the information reported on the packaging. Overall, environmentally sustainable packaging, through eco-labels constructed ad hoc for consumer needs, could represent a useful tool in the hands of food companies to encourage consumers to embrace the principles of environmental sustainability and direct them to make environmentally sustainable choices and actions.

The present study provided a theoretical contribution to the understanding of the reasons underlying the attitude–behavior gap that are useful to predict consumers’ actions and guide them towards more sustainable environmental choices in order to narrow it. Contrary to previous works that analyzed only a few variables that may influence consumer choices, this study instead contemporaneously examined as many variables as possible, from an environmental sustainability perspective It also analyzed the relationship and synergy between socio-demographic characteristics, awareness, behavioral choices and the influence of eco-labels on consumer decision making, in order to assess consumers in their entirety. This original analysis of possible sustainable consumer profiles can help to implement strategies to promote more environmentally responsible consumption in society and enable the development of best practices and policy recommendations for new and emerging market segments from an environmental sustainability perspective. Therefore, the outcomes presented here could help guide both future research and companies operating in the food industry to understand what could be the most suitable communication model through environmentally sustainable packaging that is able to entice more and more consumers to make sustainable choices that safeguard the environment.

## Figures and Tables

**Table 1:** Effect of socio-demographic characteristics on consumers’ awareness (*n* = 646).

Consumers’ Awareness Variables	Total	Gender *			Age Class					Educational Level				
		Female (64.4%)	Male (35.6%)	*p*	18–30 (42.1%)	31–45 (19.8%)	46–60 (29.6%)	61–80 (8.5%)	*p*	Primary/ Lower Secondary School (5.0%)	Upper Secondary School (35.1%)	Bachelor’s Degree (23.7%)	Master’s/ Post/Doctoral Degree (36.2%)	*p*
What participants know														
Circular economy	**5.7 ^B^**	5.8 ^a,B^	5.7 ^a,A^	0.501	5.9 ^a,A^	5.6 ^a,B^	5.7 ^a,B^	5.5 ^a,AB^	0.075	**5.0 ^b,A^**	**5.7 ^a,B^**	**5.7 ^ab,B^**	**5.9 ^a,B^**	**0.014**
Food waste	**6.1 ^A^**	**6.2 ^a,A^**	**5.9 ^b,A^**	**0.012**	6.1 ^a,A^	6.3 ^a,A^	6.2 ^a,A^	5.9 ^a,AB^	0.962	**5.4 ^b,A^**	**6.0 ^b,AB^**	**6.2 ^ab,A^**	**6.4 ^a,A^**	**0.003**
Packaging material	**6.1 ^A^**	**6.2 ^a,A^**	**6.0 ^b,A^**	**0.002**	6.1 ^a,A^	6.2 ^a,C^	6.2 ^a,A^	6.0 ^a,A^	0.767	**5.7 ^b,A^**	**6.1 ^a,A^**	**6.1 ^ab,A^**	**6.2 ^a,A^**	**0.043**
Symbols index	**1.9 ^D^**	2.0 ^a,D^	1.9 ^a,C^	0.418	**2.0 ^a,C^**	**2.2 ^a,D^**	**1.8 ^ab,D^**	**1.3 ^b,C^**	**0.024**	**1.4 ^b,B^**	**1.7 ^b,D^**	**2.2 ^a,D^**	**2.1 ^a,D^**	**0.003**
Packaging and food waste are related	**5.1 ^C^**	5.1 ^a,C^	5.0 ^a,B^	0.298	5.0 ^a,B^	5.1 ^a,C^	5.0 ^a,C^	5.4 ^a,B^	0.194	4.8 ^a,A^	5.1 ^a,C^	5.0 ^a,C^	5.1 ^a,C^	0.518
*p*	**<0.0001**	**<0.0001**	**<0.0001**		**<0.0001**	**<0.0001**	**<0.0001**	**<0.0001**		**<0.0001**	**<0.0001**	**<0.0001**	**<0.0001**	
What participants think makes packaging sustainable														
Nanotechnologies	**4.4 ^E^**	4.4 ^a,E^	4.4 ^a,D^	0.817	4.4 ^a,D^	4.3 ^a,D^	4.4 ^a,E^	4.3 ^a,C^	0.679	4.7 ^a,B^	4.4 ^a,D^	4.2 ^a,D^	4.4 ^a,D^	0.250
Regenerated materials	**5.9 ^C^**	6.0 ^a,C^	5.8 ^a,B^	0.125	6.0 ^a,B^	5.8 ^a,B^	5.9 ^a,C^	5.9 ^a,A^	0.383	5.8 ^a,A^	6.0 ^a,B^	5.8 ^a,B^	5.9 ^a,B^	0.142
Smart/active function	**5.4 ^D^**	**5.5 ^a,D^**	**5.2 ^b,C^**	**0.033**	5.4 ^a,C^	5.4 ^a,C^	5.4 ^a,D^	5.3 ^a,B^	0.847	5.6 ^a,A^	5.4 ^a,C^	5.4 ^a,C^	5.3 ^a,C^	0.449
Packaging reduction	**6.1 ^B^**	**6.2 ^a,B^**	**6.0 ^b,AB^**	**0.047**	6.0 ^a,B^	6.2 ^a,A^	6.2 ^a,B^	6.2 ^a,A^	0.269	5.9 ^a,A^	6.0 ^a,B^	6.1 ^a,A^	6.2 ^a,A^	0.145
Produces no waste and is 100% reusable	**6.3 ^A^**	6.4 ^a,A^	6.3 ^a,A^	0.126	6.3 ^a,A^	6.3 ^a,A^	6.4 ^a,A^	6.3 ^a,A^	0.588	6.1 ^a,A^	6.4 ^a,A^	6.2 ^a,A^	6.4 ^a,A^	0.176
*p*	**<0.0001**	**<0.0001**	**<0.0001**		**<0.0001**	**<0.0001**	**<0.0001**	**<0.0001**		**<0.0001**	**<0.0001**	**<0.0001**	**<0.0001**	

Mean values in bold and different lowercase letters in each row for each socio-demographic characteristic indicate statistically significant differences (*t*-test for gender; Tukey’s test for age and educational level; *p* < 0.05). Different capital letters in each column indicate statistically significant different mean values (Tukey’s test, *p* < 0.05). * Only individuals who declared a female or male gender were included in the analysis (*n* = 637).

**Table 2:** Effect of socio-demographic characteristics on consumer behavior (*n* = 646).

Sustainable Behavior Variables	Total	Gender *			Age Class					Educational Level				
		Female (64.4%)	Male (35.6%)	*p*	18–30 (42.1%)	31–45 (19.8%)	46–60 (29.6%)	61–80 (8.5%)	*p*	Primary/ Lower Secondary School (5.0%)	Upper Secondary School (35.1%)	Bachelor’s Degree (23.7%)	Master’s/ Post/ Doctoral Degree (36.2%)	*p*
I buy products in bulk	**5.2 ^D^**	**5.3 ^a,D^**	**5.0 ^b,CD^**	**0.018**	5.2 ^a,D^	5.1 ^a,DE^	5.2 ^a,DE^	5.3 ^a,CDE^	0.483	4.8 ^a,C^	5.3 ^a,CD^	5.2 ^a,DE^	5.1 ^a,DE^	0.044
I try to buy products that have less packaging	**5.6 ^C^**	**5.7 ^a,C^**	**5.3 ^b,C^**	**<0.0001**	5.5 ^a,C^	5.5 ^a,C^	5.7 ^a,C^	5.8 ^a,BCD^	0.011	5.2 ^a,BC^	5.5 ^a,C^	5.7 ^a,BC^	5.7 ^a,C^	0.009
I reduce the purchase of food in plastic packaging	**5.4 ^C^**	**5.6 ^a,C^**	**5.1 ^b,CD^**	**<0.0001**	**5.4 ^ab,CD^**	**5.3 ^b,CD^**	**5.5 ^ab,CD^**	**5.8 ^a,BC^**	**0.008**	5.4 ^a,BC^	5.5 ^a,D^	5.5 ^a,CD^	5.3 ^a,D^	0.205
I reuse the packaging of the products I buy	**5.1 ^D^**	**5.3 ^a,D^**	**4.8 ^b,DE^**	**<0.0001**	5.1 ^a,DE^	5.0 ^a,DE^	5.3 ^a,D^	5.2 ^a,DE^	0.070	5.2 ^a,BC^	5.3 ^a,CD^	5.3 ^a,CD^	5.0 ^a,EF^	0.031
I pay attention to separate waste collection	**6.6 ^A^**	**6.6 ^a,A^**	**6.5 ^b,A^**	**0.021**	**6.5 ^b,A^**	**6.6 ^ab,A^**	**6.7 ^a,A^**	**6.7 ^ab,A^**	**0.012**	**6.3 ^b,A^**	**6.5 ^ab,A^**	**6.6 ^ab,A^**	**6.7 ^a,A^**	**0.011**
I prefer to buy products whose packaging allows a longer shelf life	**4.8 ^E^**	4.8 ^a,E^	4.9 ^a,DE^	0.665	4.8 ^a,EF^	4.7 ^a,E^	4.8 ^a,F^	5.3 ^a,CDE^	0.119	5.2 ^a,BC^	4.8 ^a,E^	4.9 ^a,EF^	4.8 ^a,F^	0.524
I read the description of the packaging	**5.1 ^D^**	5.2 ^a,D^	5.0 ^a,DE^	0.062	5.2 ^a,D^	5.2 ^a,CD^	5.0 ^a,EF^	5.3 ^a,CDE^	0.327	5.0 ^a,BC^	5.0 ^a,DE^	5.3 ^a,DE^	5.1 ^a,DE^	0.364
I usually buy products from companies whose environmental sustainability values I know	**4.8 ^E^**	4.8 ^a,E^	4.6 ^a,E^	0.074	4.6 ^a,F^	4.7 ^a,E^	4.9 ^a,F^	5.0 ^a,E^	0.057	4.7 ^a,C^	4.8 ^a,E^	4.8 ^a,F^	4.7 ^a,F^	0.415
I reduce food waste	**6.0 ^B^**	6.0 ^a,B^	6.0 ^a,B^	0.623	5.9 ^a,B^	6.1 ^a,B^	6.1 ^a,B^	6.2 ^aAB^	0.011	5.7 ^a,AB^	6.0 ^a,B^	6.1 ^a,B^	6.0 ^a,B^	0.071
*p*	**<0.0001**	**<0.0001**	**<0.0001**		**<0.0001**	**<0.0001**	**<0.0001**	**<0.0001**		**<0.0001**	**<0.0001**	**<0.0001**	**<0.0001**	

Mean values in bold and different letters in each row for each socio-demographic characteristic indicate statistically significant differences (*t*-test for gender; Tukey’s test for age and educational level; *p* < 0.05). Different capital letters in each column indicate statistically significant different mean values (Tukey’s test, *p* < 0.05). * Only individuals who declared a female or male gender were included in the analysis (*n* = 637).

**Table 3:** Effect of socio-demographic characteristics on consumers’ expectations (*n* = 646).

Expectation Variables	Total	Gender *			Age Class					Educational Level				
		Female (64.4%)	Male (35.6%)	*p*	18–30 (42.1%)	31–45 (19.8%)	46–60 (29.6%)	61–80 (8.5%)	*p*	Primary/Lower Secondary School (5.0%)	Upper Secondary School (35.1%)	Bachelor’s Degree (23.7%)	Master’s/Post/ Doctoral Degree (36.2%)	*p*
What is important to be on the labels														
Indication of packaging materials	**5.9 ^C^**	5.9 ^a,C^	5.8 ^a,C^	0.501	5.9 ^a,C^	5.7 ^a,B^	5.9 ^a,D^	6.0 ^a,BCD^	0.101	**5.4 ^b,C^**	**5.8 ^ab,D^**	**6.0 ^a,B^**	**5.9 ^a,C^**	**0.012**
Indication of the type of collection	**6.4 ^B^**	**6.5 ^a,AB^**	**6.3 ^b,B^**	**0.019**	6.4 ^a,AB^	6.3 ^a,A^	6.4 ^a,B^	6.4 ^a,AB^	0.150	**5.8 ^b,ABC^**	**6.3 ^a,BC^**	**6.5 ^a,A^**	**6.4 ^a,B^**	**0.0002**
Symbols relating to the sustainability of the packaging	**5.9 ^C^**	5.9 ^a,C^	5.8 ^a,C^	0.148	**5.8 ^b,CD^**	**5.7 ^b,B^**	**6.1 ^a,C^**	**6.0 ^ab,CD^**	**0.0001**	5.7 ^a,BC^	6.0 ^a,D^	5.9 ^a,B^	5.9 ^a,C^	0.163
Narrative elements that indicate the type of packaging	**5.2 ^E^**	**5.3 ^a,D^**	**5.1 ^b,D^**	**0.042**	**5.1 ^b,E^**	**5.1 ^ab,C^**	**5.5 ^a,E^**	**5.3 ^ab,E^**	**0.011**	**5.3 ^ab,C^**	**5.4 ^a,E^**	**5.2 ^ab,C^**	**5.1 ^b,D^**	**0.039**
Packaging ecological footprint	**5.7 ^D^**	**5.8 ^a,C^**	**5.6 ^b,C^**	**0.044**	5.7 ^a,D^	5.6 ^a,B^	5.9 ^a,D^	5.7 ^a,DE^	0.040	5.4 ^a,C^	5.8 ^a,D^	5.8 ^a,B^	5.7 ^a,C^	0.104
Country of origin of the food product	**6.4 ^B^**	**6.5 ^a,AB^**	**6.3 ^b,B^**	**0.026**	6.3 ^a,B^	6.4 ^a,A^	6.5 ^a,AB^	6.4 ^a,AB^	0.143	6.1 ^a,AB^	6.4 ^a,AB^	6.3 ^a,A^	6.4 ^a,AB^	0.124
Nutritional values of the food product	**6.3 ^B^**	6.3 ^a,B^	6.2 ^a,B^	0.091	6.3 ^a,B^	6.2 ^a,A^	6.3 ^a.BC^	6.2 ^a,ABC^	0.325	**5.8 ^b,ABC^**	**6.2 ^ab,C^**	**6.3 ^ab,A^**	**6.4 ^a,B^**	**0.007**
Expiration date of the food product	**6.6 ^A^**	6.6 ^a,A^	6.6 ^a,A^	0.818	6.6 ^ab,A^	6.5 ^b,A^	6.7 ^a,A^	6.5 ^ab,A^	0.012	6.3 ^a,A^	6.6 ^a,A^	6.5 ^a,A^	6.7 ^a,A^	0.065
*p*	**<0.0001**	**<0.0001**	**<0.0001**		**<0.0001**	**<0.0001**	**<0.0001**	**<0.0001**		**<0.0001**	**<0.0001**	**<0.0001**	**<0.0001**	
How the eco-labels could be improved														
Clearer and larger symbols	**5.8 ^B^**	5.9 ^a,B^	5.8 ^a,B^	0.645	**5.6 ^b,B^**	**5.7 ^b,B^**	**6.1 ^a,BC^**	**6.3 ^a,A^**	**<0.0001**	6.1 ^a,A^	5.9 ^a,B^	5.7 ^a,C^	5.8 ^a,B^	0.220
Description of symbols	**6.2 ^A^**	**6.3 ^a,A^**	**6.1 ^b,A^**	**0.024**	6.1 ^a;A^	6.1 ^a,A^	6.3 ^a,AB^	6.2 ^a,AB^	0.168	6.2 ^a,A^	6.2 ^a,A^	6.2 ^a,AB^	6.2 ^a,A^	0.955
Enviromental impact phrases	**5.4 ^D^**	5.4 ^a,D^	5.3 ^a,C^	0.245	**5.1 ^b,C^**	**5.1 ^b,C^**	**5.9 ^a,C^**	**5.8 ^a,BC^**	**<0.0001**	5.6 ^a,AB^	5.5 ^a,C^	5.2 ^a,D^	5.3 ^a,C^	0.692
More details about the materials that make up the packaging	**5.8 ^B^**	5.9 ^a,B^	5.8 ^a,B^	0.132	**5.8 ^ab,B^**	**5.7 ^b,B^**	**6.0 ^ab,C^**	**6.0 ^a,AB^**	**0.008**	5.7 ^a,AB^	5.9 ^a,B^	5.9 ^a,BC^	5.8 ^a,B^	0.206
More details on how to recycle	**6.2 ^A^**	**6.3 ^a,A^**	**6.1 ^b,A^**	**0.004**	6.3 ^a,A^	6.1 ^a,A^	6.4 ^a,A^	6.1 ^a,AB^	0.042	6.3 ^a,A^	6.2 ^a,A^	6.3 ^a,A^	6.3 ^a,A^	0.596
QR codes or digital tools	**5.6 ^C^**	5.6 ^a,C^	5.6 ^a,BC^	0.539	5.7 ^a,B^	5.4 ^a,BC^	5.6 ^a,D^	5.3 ^a,C^	0.241	5.3 ^a,B^	5.6 ^a,C^	5.8 ^a,C^	5.5 ^a,C^	0.119
*p*	**<0.0001**	**<0.0001**	**<0.0001**		**<0.0001**	**<0.0001**	**<0.0001**	**<0.0001**		**0.001**	**<0.0001**	**<0.0001**	**<0.0001**	

Mean values in bold and different letters in each row for each socio-demographic characteristic indicate statistically significant differences (*t*-test for gender; Tukey’s test for age and educational level; *p* < 0.05). Different capital letters in each column indicate statistically significant different mean values (Tukey’s test, *p* < 0.05). * Only individuals who declared a female or male gender were included in the analysis (*n* = 637).

**Table 4:** Effect of the group on consumers’ awareness, behavior and expectations (*n* = 646).

Variables	Group 1 “More Sustainable—Packaging-Role-Oriented” (28.2%)	Group 2 “More Sustainable—Packaging Minimizers” (26.2%)	Group 3 “Less Sustainable” (21.2%)	Group 4 “Medium Sustainable” (24.5%)	*p*
What participants know					
Circular economy	**6.0 ^a,B^**	**5.9 ^a,B^**	**5.4 ^b,B^**	**5.5 ^b,B^**	**<0.0001**
Food waste	6.2 ^a,AB^	6.2 ^a,AB^	5.9 ^a,A^	6.1 ^a,A^	0.2472
Packaging material	**6.4 ^a,A^**	**6.6 ^a,A^**	**5.5 ^c,B^**	**5.9 ^b,AB^**	**<0.0001**
Symbols index	**2.2 ^a,D^**	**2.1 ^ab,D^**	**1.6 ^c,D^**	**1.8 ^bc,D^**	**0.0002**
Packaging and food waste are related	**5.6 ^a,C^**	**5.3 ^a,C^**	**4.5 ^b,C^**	**4.6 ^b,C^**	**<0.0001**
*p*	**<0.0001**	**<0.0001**	**<0.0001**	**<0.0001**	
What participants think makes packaging sustainable					
Nanotechnologies	**4.7 ^a,D^**	**4.4 ^ab,D^**	**4.1 ^b,D^**	**4.1 ^b,C^**	**<0.0001**
Regenerated materials	**6.1 ^a,B^**	**6.0 ^a,B^**	**5.5 ^b,B^**	**5.9 ^a,A^**	**<0.0001**
Smart/active function	**5.6 ^a,C^**	**5.7 ^a,C^**	**4.9 ^b,C^**	**5.2 ^b,B^**	**<0.0001**
Packaging reduction	**6.4 ^a,A^**	**6.5 ^a,A^**	**5.6 ^b,AB^**	**5.9 ^b,A^**	**<0.0001**
Produces no waste and is 100% reusable	**6.5 ^a,A^**	**6.6 ^a,A^**	**6.0 ^b,A^**	**6.2 ^b,A^**	**<0.0001**
*p*	**<0.0001**	**<0.0001**	**<0.0001**	**<0.0001**	
Sustainable behavior					
I buy products in bulk	**5.5 ^b,E^**	**6.1 ^a,CD^**	**4.8 ^c,C^**	**4.1 ^d,E^**	**<0.0001**
I try to buy products that have less packaging	**6.2 ^b,BC^**	**6.5 ^a,B^**	**4.7 ^c,C^**	**4.7 ^c,CD^**	**<0.0001**
I reduce the purchase of food in plastic packaging	**6.0 ^b,CD^**	**6.4 ^a,BC^**	**4.6 ^c,C^**	**4.4 ^c,DE^**	**<0.0001**
I reuse the packaging of the products I buy	**5.8 ^a,D^**	**5.9 ^a,D^**	**4.5 ^b,CD^**	**4.3 ^b,E^**	**<0.0001**
I pay attention to separate waste collection	**6.9 ^a,A^**	**6.8 ^ab,A^**	**5.9 ^c,A^**	**6.6 ^b,A^**	**<0.0001**
I prefer to buy products whose packaging allows a longer shelf life	**5.9 ^a,CD^**	**3.7 ^d,F^**	**4.0 ^c,E^**	**5.5 ^b,B^**	**<0.0001**
I read the description of the packaging	**6.0 ^a,CD^**	**5.3 ^b,E^**	**3.7 ^c,E^**	**5.0 ^b,C^**	**<0.0001**
I usually buy products from companies whose environmental sustainability values I know	**5.3 ^a,E^**	**5.1 ^a,E^**	**4.1 ^b,DE^**	**4.3 ^b,DE^**	**<0.0001**
I reduce food waste	**6.3 ^a,B^**	**6.4 ^a,B^**	**5.3 ^c,B^**	**5.8 ^b,B^**	**<0.0001**
*p*	**<0.0001**	**<0.0001**	**<0.0001**	**<0.0001**	
What is important to be on the labels					
Indication of packaging material	**6.3 ^a,BC^**	**6.2 ^a,C^**	**5.1 ^c,C^**	**5.7 ^b,C^**	**<0.0001**
Indication of the type of collection	**6.7 ^a,A^**	**6.6 ^a,A^**	**5.9 ^c,B^**	**6.3 ^b,B^**	**<0.0001**
Symbols relating to the sustainability of the packaging	**6.3 ^a,C^**	**6.2 ^a,C^**	**5.3 ^b,C^**	**5.6 ^b,C^**	**<0.0001**
Narrative elements that indicate the type of packaging	**5.8 ^a,D^**	**5.6 ^a,D^**	**4.5 ^c,D^**	**4.9 ^b,D^**	**<0.0001**
Packaging ecological footprint	**6.1 ^a,C^**	**6.2 ^a,C^**	**5.0 ^c,C^**	**5.4 ^b,C^**	**<0.0001**
Country of origin of the food product	**6.5 ^ab,A^**	**6.6 ^a,A^**	**6.1 ^c,AB^**	**6.3 ^bc,B^**	**<0.0001**
Nutritional values of the food product	**6.5 ^a,AB^**	**6.4 ^a,BC^**	**5.9 ^b,B^**	**6.3 ^a,B^**	**<0.0001**
Expiration date of the food product	**6.7 ^a,A^**	**6.5 ^ab,AB^**	**6.4 ^b,A^**	**6.7 ^a,A^**	**0.0001**
*p*	**<0.0001**	**<0.0001**	**<0.0001**	**<0.0001**	
How the eco-label could be improved					
Clearer and larger symbols	**6.1 ^a,B^**	**6.0 ^ab,BC^**	**5.4 ^c,B^**	**5.8 ^b,B^**	**<0.0001**
Description of symbols	**6.4 ^a,A^**	**6.3 ^a,AB^**	**5.8 ^b,A^**	**6.2 ^a,A^**	**<0.0001**
Environmental impact phrases	**5.7 ^a,C^**	**5.6 ^ab,D^**	**4.8 ^c,C^**	**5.3 ^b,C^**	**<0.0001**
More details about the materials that make up the packaging	**6.1 ^a,B^**	**6.0 ^ab,BC^**	**5.4 ^c,B^**	**5.7 ^b,B^**	**<0.0001**
More details on how to recycle	**6.5 ^a,A^**	**6.4 ^a,A^**	**5.9 ^b,A^**	**6.1 ^b,A^**	**<0.0001**
QR codes or digital tools	**5.9 ^a,BC^**	**5.7 ^ab,CD^**	**5.2 ^c,B^**	**5.4 ^bc,C^**	**<0.0001**
*p*	**<0.0001**	**<0.0001**	**<0.0001**	**<0.0001**	

Mean values in bold and different letters in each row for each socio-demographic characteristic indicate statistically significant differences (*t*-test for gender; Tukey’s test for age and educational level; *p* < 0.05). Different capital letters in each column indicate statistically significant different mean values (Tukey’s test, *p* < 0.05).

**Table 5:** Socio-demographic characteristics of the four identified groups of participants (*n* = 646).

Variables	Total	Group 1 “More Sustainable—Packaging-Role-Oriented” (28.2%)	Group 2 “More Sustainable—Packaging Minimizers” (26.2%)	Group 3 “Less Sustainable” (21.2%)	Group 4 “Medium Sustainable” (24.5%)	χ^2^	*p*
Gender *						18.56	0.0003
Females	64.4	**71.9 >**	**71.9 >**	**55.2 <**	**55.7 <**		
Males	35.6	**28.1 <**	**28.1 <**	**44.8 >**	**44.3 >**		
*Age*						16.09	0.065
18–30	42.1	41.6	**35.3 <**	**52.2 >**	39.9		
31–45	19.8	15.7	22.8	17.2	24.1		
46–60	29.6	30.3	32.9	25.4	29.1		
61–80	8.5	12.4	9.0	5.2	7.0		
Educational level						10.85	0.286
Primary/Lower secondary school	5.0	4.5	3.6	3.7	8.2		
Upper secondary school	35.1	34.3	34.7	42.5	30.4		
Bchelor’s degree	23.7	27.5	24.6	20.9	21.5		
Master’s/Post/Doctoral degree	36.2	33.7	37.1	32.8	39.9		

* Only individuals who declared a female or male gender were included in the analysis (*n* = 637). < and > indicate that the observed value is significantly lower or higher than the expected theoretical value and mean values in bold in each row for each socio-demographic characteristic indicate statistically significant differences (χ^2^ per cell significant for α = 0.05; Fisher’s exact probability test, *p* < 0.05).

## Data Availability

The data presented in this study are available on request from the corresponding author. The data are not publicly available due to privacy.

## Supplementary Materials

The following supporting information can be downloaded at: https://www.mdpi.com/article/10.3390/foods11162388/s1, The English version of questionnaire.
