# Supplementary material for: Consumers’ Awareness, Behavior and Expectations for Food Packaging Environmental Sustainability: Influence of Socio-Demographic Characteristics
Source: Foods. 2022 Aug 9;11(16):2388. doi: 10.3390/foods11162388 (PMC9407116; doi:10.3390/foods11162388)
Supplement: Supplementary file 1 [file foods-11-02388-s001.zip › foods-1837826-supplementary.pdf]

ITALIAN VERSION OF THE QUESTIONNAIRE

Ti ringraziamo per aver accettato di partecipare a questo studio condotto dall'Università di Scienze Gastronomiche di Pollenzo.
Il questionario richiede circa 10 minuti per essere completato.
Ti verrà chiesto di rispondere a domande sulle tue reazioni in merito al packaging alimentare dal punto di vista della sostenibilità. Non ci sono risposte corrette o errate per cui non preoccuparti, prenditi il tempo necessario e rispondi nella maniera più naturale e istintiva possibile.
La partecipazione è completamente volontaria e puoi ritirarti in qualsiasi momento senza dare motivazioni. Le tue risposte saranno memorizzate in modo anonimo e analizzate in modo aggregato.
Per maggiori informazioni contattare m.molino@studenti.unisg.it.
Ai sensi del Regolamento (UE) 2016/679 approvato dal Parlamento Europeo e dal Consiglio e ai sensi della legge italiana n. 101/18 del 10 agosto 2018 e sue modifiche pubblicate sulla Gazzetta Ufficiale n. 205 del 04 settembre 2018 (tutela delle persone e di altri soggetti rispetto al trattamento dei dati personali) il trattamento delle informazioni da Lei fornite si svolgerà nel rispetto dei principi di correttezza, liceità e tutela della Sua privacy e dei Suoi diritti.
Ho preso nota delle condizioni di cui sopra e continuando acconsento a partecipare in modo anonimo allo studio.

Genere

- ○ Maschio
- ○ Femmina
- ○ Altro
- ○ Preferisco non dichiararlo

Età

▼ 18 (18) ... 100 (100)

Nazionalità

▼ Afghanistan (1) ... Zimbabwe (1357)

Titolo di studio

- ○ Licenza elementare
- ○ Licenza media
- ○ Diploma
- ○ Laurea triennale
- ○ Laurea specialistica
- ○ Dottorato
- ○ Specializzazione/Master

Indica il significato dei seguenti simboli

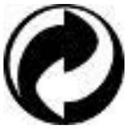

- ○ Il materiale è realizzato con materiale riciclato
- ○ Il materiale può essere riciclato
- ○ Il produttore ha fornito un contributo finanziario al recupero o al riciclaggio dei materiali
- ○ Il materiale è realizzato con materiali bio-based
- ○ Il materiale è compostabile
- ○ Non so cosa significhi il simbolo

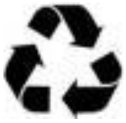

- ○ Il materiale è realizzato con materiale riciclato
- ○ Il materiale può essere riciclato
- ○ Il produttore ha fornito un contributo finanziario al recupero o al riciclaggio dei materiali
- ○ Il materiale è realizzato con materiali bio-based
- ○ Il materiale è compostabile
- ○ Non so cosa significhi il simbolo

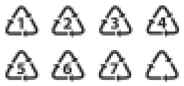

- ◯ Il livello di materiale riciclato utilizzato
- ◯ La regione di origine
- ◯ Il tipo di plastica
- ◯ Il livello di biodegradabilità
- ◯ Quanto il produttore ha contribuito al recupero o al riciclaggio dei materiali
- ◯ Non so cosa significhi il simbolo

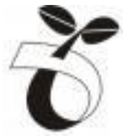

- ◯ Il materiale è realizzato con materiale riciclato
- ◯ Il materiale può essere riciclato
- ◯ Il produttore ha fornito un contributo finanziario al recupero o al riciclaggio dei materiali
- ◯ Il materiale è realizzato con materiali biobased
- ◯ Il materiale è compostabile
- ◯ Non so cosa significhi il simbolo

○ Il materiale proviene dall'Unione Europea

○ Il materiale ha un alto contenuto di materiale riciclato

○ Il materiale è compostabile

○ Il produttore ha fornito un contributo finanziario al recupero o al riciclaggio dei materiali

○ Il materiale è realizzato con legno proveniente da fonti sostenibili

○ Non so cosa significhi il simbolo

○ Il produttore ha fornito un contributo finanziario al recupero o al riciclaggio dei materiali

○ L'imballaggio è sicuro, prodotto con energia rinnovabile, basso utilizzo di acqua e il materiale è riutilizzabile o compostabile

○ L'imballaggio può essere riciclato

○ L'imballaggio è certificato per durare a lungo

○ L'imballaggio è composto al 100% da materiale riciclato

○ Non so cosa significhi il simbolo

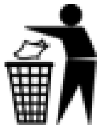

- Il materiale può essere compostato in casa
- Il materiale può essere riciclato
- Il materiale non può essere riciclato
- Non sporcare
- Il materiale è realizzato con materiale riciclato
- Non so cosa significhi il simbolo

Sono a conoscenza del concetto "economia circolare"

- Completamente in disaccordo
- In disaccordo
- Abbastanza in disaccordo
- Né in accordo né in disaccordo
- Abbastanza d'accordo
- D'accordo
- Completamente d'accordo

Sono a conoscenza del concetto "spreco alimentare"

- Completamente in disaccordo
- In disaccordo
- Abbastanza in disaccordo
- Né in accordo né in disaccordo
- Abbastanza d'accordo
- D'accordo
- Completamente d'accordo

Penso che sia importante tenere in considerazione il materiale del packaging nel momento di acquisto

- Completamente in disaccordo
- In disaccordo
- Abbastanza in disaccordo
- Né in accordo né in disaccordo
- Abbastanza d'accordo
- D'accordo
- Completamente d'accordo

Penso che il packaging di un prodotto e lo spreco alimentare siano correlati

- Completamente in disaccordo
- In disaccordo
- Abbastanza in disaccordo

◯ Né in accordo né in disaccordo

◯ Abbastanza d'accordo

◯ D'accordo

◯ Completamente d'accordo

Penso che un packaging sostenibile da un punto di vista ambientale si contraddistingua

	Completamente in disaccordo	In disaccordo	Abbastanza in disaccordo	Né in accordo né in disaccordo	Abbastanza d'accordo	D'accordo	Completamente d'accordo
Utilizzo di nanotecnologie	◯	◯	◯	◯	◯	◯	◯
Realizzato con materiali rigenerati	◯	◯	◯	◯	◯	◯	◯
Funzione intelligente/attiva	◯	◯	◯	◯	◯	◯	◯
Riduzione del packaging stesso	◯	◯	◯	◯	◯	◯	◯
Non produce scarti ed è riutilizzabile al 100%	◯	◯	◯	◯	◯	◯	◯

per

Indica quanto sei d'accordo con le seguenti affermazioni

	Completamente in disaccordo	In disaccordo	Abbastanza in disaccordo	Né in accordo né in disaccordo	Abbastanza d'accordo	D'accordo	Completamente d'accordo
Acquisto prodotti sfusi	○	○	○	○	○	○	○
Cerco di acquistare prodotti che hanno meno packaging	○	○	○	○	○	○	○
Riutilizzo il packaging dei prodotti che acquisto	○	○	○	○	○	○	○
Preferisco acquistare prodotti il cui packaging consenta una maggiore conservabilità	○	○	○	○	○	○	○
Leggo la descrizione del packaging	○	○	○	○	○	○	○

Indica quanto sei d'accordo con le seguenti affermazioni

	Completamente in disaccordo	In disaccordo	Abbastanza in disaccordo	Né in accordo né in disaccordo	Abbastanza d'accordo	D'accordo	Completamente d'accordo
Riduco l'acquisto di alimenti in confezioni di plastica	○	○	○	○	○	○	○
Faccio attenzione alla raccolta differenziata	○	○	○	○	○	○	○
Solitamente acquisto prodotti di aziende di cui conosco i valori di sostenibilità ambientale	○	○	○	○	○	○	○
Riduco gli scarti alimentari	○	○	○	○	○	○	○

Per me è importante che sulla confezione sia riportata

	Completamente in disaccordo	In disaccordo	Abbastanza in disaccordo	Né in accordo né in disaccordo	Abbastanza d'accordo	D'accordo	Completamente d'accordo
Indicazione del materiale del packaging	○	○	○	○	○	○	○
Indicazione sulla tipologia di raccolta del packaging	○	○	○	○	○	○	○
Simboli relativi alla sostenibilità ambientale del packaging	○	○	○	○	○	○	○
Elementi narrativi che raccontano la tipologia di packaging	○	○	○	○	○	○	○
Impronta ecologica del packaging	○	○	○	○	○	○	○
Paese di origine del prodotto alimentare	○	○	○	○	○	○	○
Valori nutrizionali del prodotto alimentare	○	○	○	○	○	○	○
Data di scadenza del prodotto alimentare	○	○	○	○	○	○	○

Penso che le aziende potrebbero aumentare la comunicazione della sostenibilità ambientale del packaging in etichetta tramite

	Completamente in disaccordo	In disaccordo	Abbastanza in disaccordo	Né in accordo né in disaccordo	Abbastanza d'accordo	D'accordo	Completamente d'accordo
Simboli più chiari e più grandi	○	○	○	○	○	○	○
Descrizione dei simboli	○	○	○	○	○	○	○
Frasi sull'impatto ambientale	○	○	○	○	○	○	○
Maggiori dettagli sui materiali che compongono il packaging	○	○	○	○	○	○	○
Maggiori dettagli sulle modalità di riciclo	○	○	○	○	○	○	○
QR Code o strumenti digitali	○	○	○	○	○	○	○

Grazie per aver completato il sondaggio.

La risposta è stata registrata.

ENGLISH VERSION OF THE QUESTIONNAIRE

Thank you for agreeing to participate in this study conducted by the University of Gastronomic Sciences in Pollenzo. The questionnaire requires about 10 minutes to complete. You will be asked to answer questions about your opinions about food packaging from a sustainability perspective. There are no right or wrong answers so do not worry, take your time and answer as naturally and instinctively as possible. Participation is completely voluntary and you can withdraw at any time without giving reasons. Your answers will be recorded anonymously and analyzed in aggregate. For more information contact m.molino@studenti.unisg.it. According to Regulation (EU) 2016/679 approved by the European Parliament and the Council and pursuant to Italian Law No. 101/18 of August 10, 2018 and its amendments published in the Official Gazette No. 205 of September 04, 2018 (protection of the people and other subjects with regard to the processing of personal data), the processing of the information you provide will be carried out in accordance with the principles of fairness, lawfulness and protection of your privacy and your rights. I have taken note of the above conditions and by continuing I agree to participate anonymously in the study.

Gender

- ○ Male
- ○ Female
- ○ Other gender
- ○ Preferred not to report

Age

▼ 18 (18) ... 100 (100)

Nationality

▼ Afghanistan (1) ... Zimbabwe (1357)

Education level

- ○ Primary school license
- ○ Lower secondary school license
- ○ Upper secondary school diploma
- ○ Bachelor’s degree
- ○ Master’s degree
- ○ Doctoral degree
- ○ Post degree

Indicate the meaning of the symbols shown below

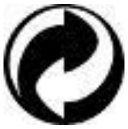

- ◯ The material is made from recycled material
- ◯ The material can be recycled
- ◯ The manufacturer has made a financial contribution to recovery or recycling of materials
- ◯ The material is made from biobased materials
- ◯ The material is compostable
- ◯ I don’t know what the symbol means

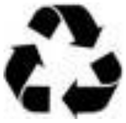

- ◯ The material is made from recycled material
- ◯ The material can be recycled
- ◯ The manufacturer has made a financial contribution to recovery or recycling of materials
- ◯ The material is made from biobased materials
- ◯ The material is compostable
- ◯ I don’t know what the symbol means

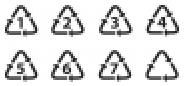

- ◯ The degree of recycled material used
- ◯ The region of origin
- ◯ The type of plastic
- ◯ The level of biodegradability
- ◯ How much the manufacturer has contributed to the recovery or recycling of materials
- ◯ I don’t know what the symbols means

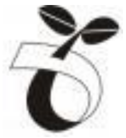

- ◯ The material is made from recycled material
- ◯ The material can be recycled
- ◯ The manufacturer has made a financial contribution to recovery or recycling of materials
- ◯ The material is made from biobased materials
- ◯ The material is compostable
- ◯ I don’t know what the symbol means

○ The material is sourced within the European Union

○ The material has a high content of recycled material

○ The material is compostable

○ The manufacturer has made a financial contribution to recovery or recycling of materials

○ The material is made from sustainably sourced wood

○ I don’t know what the symbols means

○ The manufacturer has made a financial contribution to recovery or recycling of materials

○ The packaging is safe, manufactured with renewable energy, low use of water and the material is fill reusable or compostable

○ The packaging can be recycled

○ The packaging is certified to last for a long time

○ The packaging is made from 100% recycled material

○ I don’t know what the symbols means

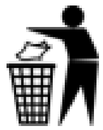

- The material can be home composted
- The material can be recycled
- The material can not be recycled
- Don't litter
- The material is made from recycled material
- I don’t know what the symbol means

I am aware of the concept of “circular economy”

- Extremely disagree
- Disagree
- Somewhat disagree
- Neither agree or disagree
- Somewhat agree
- Agree
- Extremely agree

I am aware of the concept of “food waste”

- ◯ Extremely disagree
- ◯ Disagree
- ◯ Somewhat disagree
- ◯ Neither agree or disagree
- ◯ Somewhat agree
- ◯ Agree
- ◯ Extremely agree

I think that it is important to consider packaging material when purchasing

- ◯ Extremely disagree
- ◯ Disagree
- ◯ Somewhat disagree
- ◯ Neither agree or disagree
- ◯ Somewhat agree
- ◯ Agree
- ◯ Extremely agree

I think that the food product packaging and food waste are related

- ◯ Extremely disagree
- ◯ Disagree
- ◯ Somewhat disagree

○ Neither agree or disagree

○ Somewhat agree

○ Agree

○ Extremely agree

I think that environmental sustainable packaging is distinguished by

	Extremely disagree	Disagree	Somewhat disagree	Neither agree or disagree	Somewhat agree	Agree	Extremely agree
Use of nanotechnologies	○	○	○	○	○	○	○
Made by regenerated materials	○	○	○	○	○	○	○
Smart/active function	○	○	○	○	○	○	○
Packaging reduction	○	○	○	○	○	○	○
Produces no waste and is 100% reusable	○	○	○	○	○	○	○

Indicate your level of agreement with the following statements

	Extremely disagree	Disagree	Somewhat disagree	Neither agree or disagree	Somewhat agree	Agree	Extremely agree
I buy products in bulk	○	○	○	○	○	○	○
I try to buy products that have less packaging	○	○	○	○	○	○	○
I reuse the packaging of the products I buy	○	○	○	○	○	○	○
I prefer to buy products whose packaging allows a longer shelf life	○	○	○	○	○	○	○
I read the description of the packaging	○	○	○	○	○	○	○

Indicate your level of agreement with the following statements

	Extremely disagree	Disagree	Somewhat disagree	Neither agree or disagree	Somewhat agree	Agree	Extremely agree
I reduce the purchase of food in plastic packaging	○	○	○	○	○	○	○
I pay attention to separate waste collection	○	○	○	○	○	○	○
I usually buy products from companies whose environmental sustainability values I know	○	○	○	○	○	○	○
I reduce food waste	○	○	○	○	○	○	○

For me it is important to find on food packaging

	Extremely disagree	Disagree	Somewhat disagree	Neither agree or disagree	Somewhat agree	Agree	Extremely agree
Indication of packaging materials	○	○	○	○	○	○	○
Indication of the type of collection	○	○	○	○	○	○	○
Symbols relating to the environmental sustainability of the packaging	○	○	○	○	○	○	○
Narrattive elements that tell the type of the packaging	○	○	○	○	○	○	○
Packaging ecological footprint	○	○	○	○	○	○	○
Country of origin of the food product	○	○	○	○	○	○	○
Nutritional values of the food product	○	○	○	○	○	○	○
Expiration date of the food product	○	○	○	○	○	○	○

I think that companies could increase the communication of the food packaging sustainability improving the label by means of

	Extremely disagree	Disagree	Somewhat disagree	Neither agree or disagree	Somewhat agree	Agree	Extremely agree
Clearer and larger symbols	○	○	○	○	○	○	○
Description of symbols	○	○	○	○	○	○	○
Environmental impact phrases	○	○	○	○	○	○	○
More details about the materials that make up the packaging	○	○	○	○	○	○	○
More details on how to recycle	○	○	○	○	○	○	○
QR Code or digital tools	○	○	○	○	○	○	○

Thank you for filling out the survey.

Your response has been recorded.
